# Addressing Unmet Needs in Cardiovascular Care: A Focus on Centers for Medicaid and Medicare Services' Efforts to Integrate Social Determinants of Health Into Health Care

**DOI:** 10.1016/j.jacadv.2025.101813

**Published:** 2025-05-19

**Authors:** Kardie Tobb, Ritu Thamman, Gladys Velarde, Jen Nixon, Latha Palaniappan, Adrian M. Bacong, Rishi K. Wadhera, Eric J. Brandt

**Affiliations:** a Cone Health Heart & Vascular, Greensboro, North Carolina, USA; b Cone Health Center for Health Equity, Greensboro, North Carolina, USA; c University of Pittsburgh School of Medicine, Pittsburgh, Pennsylvania, USA; d University of Florida, Jacksonville, Florida, USA; e Division of Cardiovascular Medicine, Department of Medicine, Stanford University School of Medicine, Stanford, California, USA; f Beth Israel Deaconess Medical Center, Harvard Medical School, Boston, Massachusetts, USA; g Department of Health Policy and Management, Harvard Medical School, Boston, Massachusetts, USA; h Institute for Healthcare Policy and Innovation, University of Michigan, Ann Arbor, Michigan, USA; i Division of Cardiovascular Medicine, Department of Internal Medicine, University of Michigan, Ann Arbor, Michigan, USA

**Keywords:** health equity, SDOH

Social determinants of health (SDOH) are the environmental conditions, in which people are born, live, grow, work, play, and age that impact health outcomes.^1^ SDOH relate to multiple levels of an individual's and community's sociopolitical, economic, and community contexts that influence individual-level factors (See Figure 1, based on the World Health Organization conceptual framework of action on SDOH). Differences in cardiovascular disease outcomes across sociodemographic variables are largely eliminated after accounting for SDOH.^1^ This understanding is now reflected in the incorporation of area-level SDOH within the recently developed race-agnostic American Heart Association PREVENT (Predicting Risk of cardiovascular disease EVENTs) Equations.^2^, ^3^, ^4^ The Centers for Medicaid and Medicare Services (CMS) also recognizes the importance of SDOH in their 2022 Framework for Healthy Equity and has made recommendations to standardize collection of SDOH data, integrate SDOH into billable medical care, and adjust health system reimbursements as ways to promote healthy equity. Herein we provide information about the ongoing changes with CMS that will impact the health care systems in which we practice.

**Figure 1:**
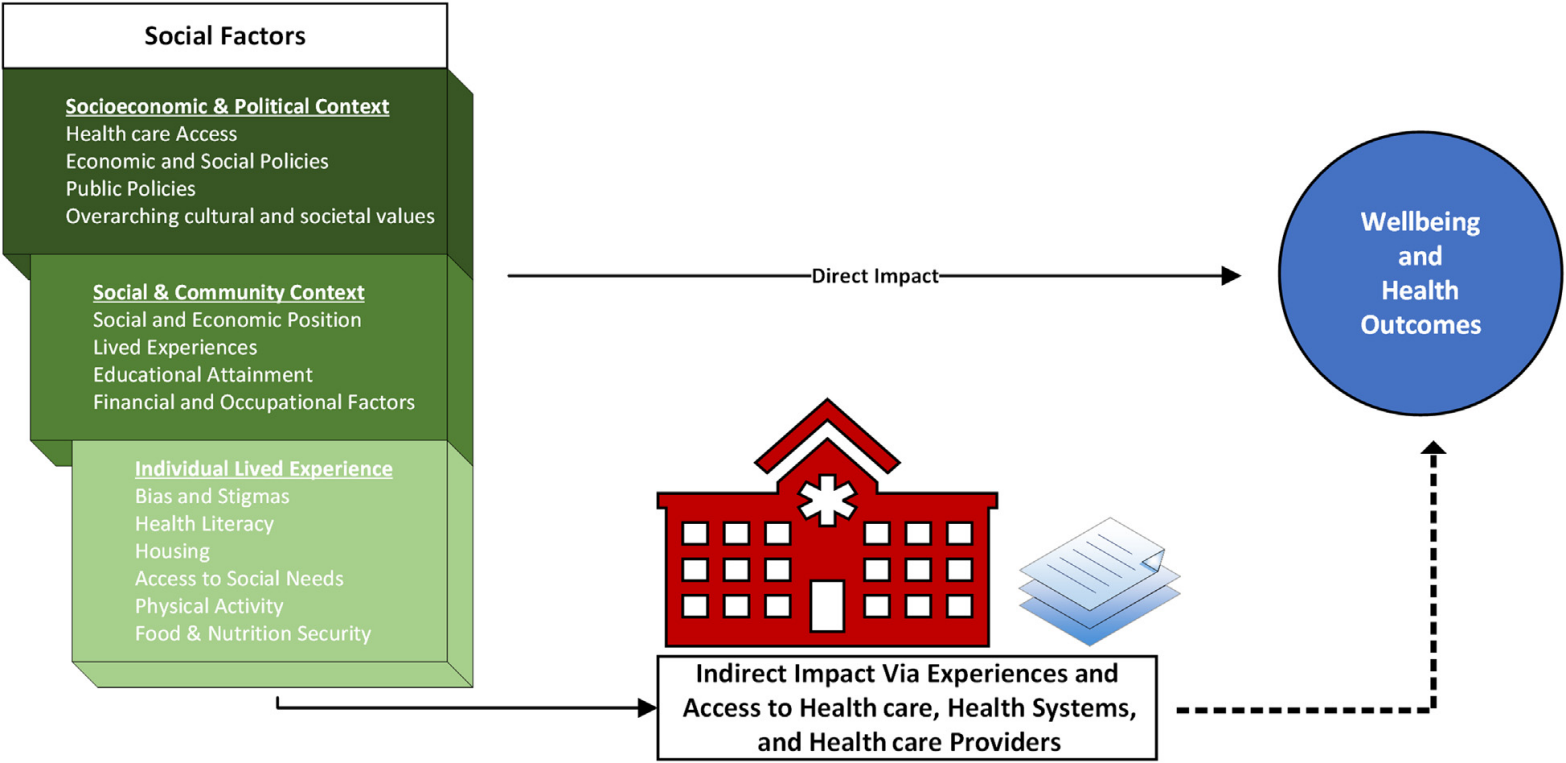
Based on the World Health Organization Conceptual Framework of Action on SDOH SDOH = social determinants of health.

Evidence-based cardiovascular treatment is underutilized and disproportionately impacts individuals based on racial and ethnic identity.^5^ For example, reports from 2008 and recent years have shown increasing prevalences of obesity (32.7% to >40.9%), diabetes (3% to >4.1%), and hypertension (9.3% to >11.5%) that is disproportionately high among young Black and Hispanic individuals. Key contributors to the disproportionate changes include unmet SDOH needs, health care professional biases, experiences of racism, clinical inertia, and systemic barriers such as underfunding, inappropriate resource allocation, and adverse health care policies.^6^ It is notable that in the current state, hospitals serving predominantly low-income and minority populations often face significant underfunding.^7^^,^^8^ This chronic under-resourcing forces hospitals to cut essential services, from preventive care to advanced lifesaving therapies, limiting patient access to technologies and treatments crucial for positive health outcomes.^8^

The current state of CMS includes national value-based programs like the Value-Based Purchasing program and Hospital Readmissions Reduction Program by CMS, which tie hospital performance on certain quality metrics to reimbursement rates to incentivize high-quality care. Many outpatient practices are required to participate in the Merit Incentive-Based Payment System, another value-based program enacted by CMS that financially rewards or penalizes practices based on quality measure performance. This system creates challenges for hospitals and practices serving disadvantaged populations, as these hospitals often find it harder to achieve high scores due to socioeconomic barriers faced by their patients, such as limited access to follow-up care, lower health literacy, and higher rates of comorbidities. Consequently, these hospitals and practices are more likely to be penalized by these CMS programs. CMS is taking action in these areas and has recognized the role of SDOH in health in several ways:

1. Z-codes: The CMS Framework for Health Equity identifies the need to expand the collection of standardized data on SDOH. There have been continued additions to International Classification of Diseases, tenth Revision, Clinical Modification “Z-codes” that allow clinicians to track SDOH from various domains. However, Z-codes have been significantly underused and do not reflect the actual burden of social needs experienced by patients.^9^
2. Health-related social needs: CMS' 2024 mandate for hospitals to screen for 5 key health-related social needs (food insecurity, housing instability, transportation needs, utility difficulty, and interpersonal safety.^10^ Furthermore, CMS has encouraged all clinicians to routinely collect information on health literacy, patient language, interpreter needs, transportation, and social isolation.^10^
3. Reimbursement: CMS also recognizes the financial support needed to address SDOH in the clinical context and added billing codes to the physician fee schedule.
  • Billing codes: Potential pitfalls of these are that patients may have additional copays when these codes are assigned:
    ○ Office visits: G0136 can be used for when SDOH are assessed and addressed in an office visit (relative value unit = 0.18).^10^
    ○ Community support: CMS also added billing codes for Community Health Integration (G0019, G0022) and Principal Illness Navigation Services (G0023, G0024, G0140, G0146), which are billed by auxiliary personnel under a physician or other practitioner. These billing codes provide a financial structure to support nonphysicians (social workers, community health workers, etc) who are needed to address SDOH.
  • The economic burden of health inequities on cardiovascular (CV) outcomes is so enormous that a health equity adjustment to hospitals through CMS' Value-Based Purchasing Program will start in 2026. It is projected to reclassify hospital bonus and penalty status and to increase payments to those hospitals caring for minority and low-income populations.^10^
4. Health equity measures: CMS recently required inpatient hospitals participating in the Inpatient Quality Reporting program to adopt health equity measures that will allow for hospitals to make a commitment to health equity as a strategic priority, collect health-related social needs data, analyze these data, and create quality improvement activities that focus on eliminating health disparities.^9^

Building on the current CMS framework, it remains important to acknowledge the uncertainty surrounding future policies. At the time of this writing, CMS has made significant strides in integrating SDOH into health care delivery. These initiatives—including standardized data collection, billing mechanisms, and value-based payment adjustments—reflect a commitment to advancing health equity. The future of these policies remains uncertain. Administrative shifts may influence funding, implementation strategies, and the prioritization of health equity initiatives within federal health care programs.

The impact of SDOH on CV health is well-established and cannot be overlooked. It remains imperative for health care professionals, institutions, and policymakers to advocate for an implementation strategy that address these determinants. Sustainable, data-driven, and patient-centered approaches must continue to be prioritized to reduce disparities and improve CV outcomes, independent of political and regulatory shifts. We are amid a realignment of health care services that seek to directly focus on the management of social factors not previously considered in clinical care. The future of these programs is uncertain, but what is clear is that health practitioners and health care systems must remain aware of their evolution to maximize opportunities to improve health outcomes that are driven by SDOH or that may have important financial implications.

## Funding support and author disclosures

The authors have reported that they have no relationships relevant to the contents of this paper to disclose.
